# Effect of Fe(III) on the positive electrolyte for vanadium redox flow battery

**DOI:** 10.1098/rsos.181309

**Published:** 2019-01-23

**Authors:** Muqing Ding, Tao Liu, Yimin Zhang, Zhenlei Cai, Yadong Yang, Yizhong Yuan

**Affiliations:** 1 School of Resource and Environmental Engineering, Wuhan University of Science and Technology, Wuhan 430081, Hubei Province, People's Republic of China; 2 State Environmental Protection Key Laboratory of Mineral Metallurgical Resources Utilization and Pollution Control, Wuhan University of Science and Technology, Wuhan 430081, Hubei Province, People's Republic of China; 3 Hubei Provincial Engineering Technology Research Center of Highly Efficient Cleaning Utilization for Shale Vanadium Resources, Wuhan University of Science and Technology, Wuhan 430081, Hubei Province, People's Republic of China; 4 Hubei Collaborative Innovation Center for Highly Efficient Utilization of Vanadium Resources, Wuhan University of Science and Technology, Wuhan 430081, Hubei Province, People's Republic of China; 5 School of Resource and Environmental Engineering, Wuhan University of Technology, Wuhan 430070, Hubei Province, People's Republic of China

**Keywords:** vanadium redox flow battery, Fe(III), thermal stability, electrochemical behaviour, competition of charge–discharge

## Abstract

It is important to study the effect of Fe(III) on the positive electrolyte, in order to provide some practical guidance for the preparation and use of vanadium electrolyte. The effect of Fe(III) on the thermal stability and electrochemical behaviour of the positive electrolyte for the vanadium redox flow battery (VRFB) was investigated. When the Fe(III) concentration was above 0.0196 mol l^−1^, the thermal stability of V(V) electrolyte was impaired, the diffusion coefficient of V(IV) species decreased from (2.06–3.33) × 10^−6^ cm^2^ s^−1^ to (1.78–2.88) × 10^−6^ cm^2^ s^−1^, and the positive electrolyte exhibited a higher electrolyte resistance and a charge transfer resistance. Furthermore, Fe(III) could result in the side reaction and capacity fading, which would have a detrimental effect on battery application. With the increase of Fe(III), the collision probability of vanadium ions with Fe(III) and the competition with the redox reaction was aggravated, which would interfere with the electrode reaction, the diffusion of vanadium ions and the performance of VRFB. Therefore, this study provides some practical guidance that it is best to bring the impurity of Fe(III) below 0.0196 mol l^−1^ during the preparation and use of vanadium electrolyte.

## 1. Introduction

Recently, with the ongoing environmental pollution and energy crisis, the vanadium redox flow battery (VRFB) has been considered as an efficient and environmentally-friendly storage unit for a wide range of applications [1,2]. VRFB stores energy in the form of electrolyte solutions rather than electrodes, which is the most obvious difference between VRFB and traditional solid secondary batteries. VRFBs are installed as the positive and negative electrolytes of energy storage materials in two liquid storage tanks, which means they have many advantages, such as high efficiency, fast response, deep discharge ability, flexible module design and long lifetime [3–5]. Because of these merits, VRFB is widely commercially applied and researched.

The vanadium electrolyte is not only an important part of the VRFB structure but also the energy storage substance of the battery, hence the energy density of VRFB is directly determined by the character of the electrolyte [6,7]. However, the poor thermal stability of the electrolyte, especially the positive electrolyte, greatly limits the concentration of active material in the electrolyte, which significantly affects the performance of VRFB [8–12]. In general, the impurity ions are thought to have a serious effect on the thermal stability of the electrolyte [13]. Thus far, researchers have reported that the electrochemical activity and kinetics of the V(IV)/V(V) redox couple could be enhanced by the addition of In^3+^ ions [14], and the Cr^3+^ could affect the electrochemical performance of the V(IV)/V(V) redox reaction, including the reaction activity, the reversibility of the electrode reaction and the diffusivity of vanadium ions [15]. A large amount of impurity ions, particularly Fe(III), are available in the intermediate product (rich vanadium liquid) of vanadium extraction from vanadium shale. Deng Zhi-gan *et al*. [16] obtained the rich vanadium liquid containing 37 g l^−1^ V and 0.6 g l^−1^ Fe by oxygen pressure acid leaching and solvent extraction. Li Xing-bin *et al*. [17] selected solvent extraction of vanadium from black shale acid leach solution (containing 5.78 g l^−1^ V_2_O_5_ and 10.86 g l^−1^ total Fe) by D2EHPA/TBP, and the rich vanadium liquid contains 26.3 g l^−1^ V_2_O_5_ and 0.72 g l^−1^ Fe. In the industrial production process, when the concentration of vanadium in rich vanadium liquid is further enriched, the impurity ions in the solution will also be enriched, so the concentration of Fe will be higher. However, vanadium oxides usually contain iron impurity because Fe(III) is hard to separate from vanadium [18–21].

Because impurity ions have a great influence on the properties of the electrolyte, it is required that the vanadium electrolyte raw materials, such as VOSO_4_ and V_2_O_5_, should be highly pure, resulting in a higher cost for VRFB production [22]. By contrast, if it is possible to use the low-purity raw materials such as rich vanadium liquid to directly prepare the electrolyte, the cost of VRFB can be greatly reduced. In addition, when the impurity ions in the electrolyte reach a certain concentration limitation, the electrolyte will lose its activity and will need to be regenerated. However, the concentration limitation of iron ions in the electrolyte is not clear and the law of the influence of the electrolyte performance is not clear. A further systematic study was carried out to determine the concentration limitation of iron ions in the electrolyte, thus providing some theoretical guidance for the regeneration treatment of the electrolyte during use. In view of that, understanding the influence of the Fe(III) impurity ions on the thermal stability and electrochemical behaviour of the electrolyte is imperative.

In this paper, we studied in detail different concentrations of Fe(III) as impurity ions for VRFB anolytes and investigated its effect on the thermal stability and electrochemical performance. Also, the influence mechanism of Fe(III) on the redox reaction was discussed. This study may provide some practical guidance for the preparation and use of vanadium electrolyte.

## 2. Experimental set-up

### 2.1. Preparation of electrolyte

The electrolyte with 1.6 mol l^−1^ VOSO_4_ and 2.8 mol l^−1^ H_2_SO_4_ presents the optimal electrochemical performance [12]. V(IV) electrolyte (1.6 M V(IV) + 2.8 M H_2_SO_4_) was prepared by dissolving VOSO_4_ in concentrated sulfuric acid. Then V(V) electrolyte (1.6 M V(V) + 2.8 M H_2_SO_4_) can be obtained in a two-compartment electrolysis cell which used V(IV) electrolyte as an anolyte and H_2_SO_4_ solution in the same concentration as the catholyte. The vanadium concentration in the electrolyte was analysed by the potentiometric redox titration method. The procedure of the redox titration method is to add the sulfur and phosphorus mixed acid, then add potassium permanganate solution to the electrolyte until the solution becomes purple, and finally, conduct a titration using a potentiometric titrator with ferrous ammonium sulfate standard solution until the potential of electrolyte changes; the vanadium concentration can be calculated through the titration volume and concentration of the standard solution [23]. A certain concentration (0.00893 mol l^−1^, 0.0161 mol l^−1^, 0.0196 mol l^−1^, 0.0232 mol l^−1^, 0.0286 mol l^−1^) of Fe(III) (Fe_2_(SO_4_)_3_^.^xH_2_O) was added into the electrolytes. (The influence of sulfate ion on the electrolytes can be ignored [13].) The total iron concentration in the electrolytes was determined by ICP-AES. The ferrous iron concentration in the electrolytes was measured using the 1,10-phenanthroline spectrophotometry [24]. The ferric iron concentration in the electrolytes was calculated by mass balance. All the chemical reagents used in the experiments were analytically pure and the solutions were prepared with deionized water.

### 2.2. Thermal stability experiment

During the stability tests, the electrolyte with different concentrations of Fe(III) was statically stored in a temperature-controlled bath at different temperatures (25°C, 40°C and 50°C). The samples were monitored regularly for the formation of precipitation and the time when a slight precipitation appeared was recorded. The solutions were filtered at regular intervals and titrated to determine the changes in vanadium concentration.

### 2.3. Electrochemical measurements

Cyclic voltammetry (CV), electrochemical impedance spectroscopy (EIS) of the V(IV) electrolyte with different amounts of Fe(III) and Linear sweep voltammetry (LSV) of 2.8 M H_2_SO_4_ solution with different concentrations of Fe(III) were performed using the CHI660 electrochemical workstation (Shanghai Chenhua Instrument Co., Ltd., China). The curves of current versus potential were recorded in a three-electrode electrochemical cell with a platinum sheet electrode (2 cm^2^) as the counter electrode, an aqueous saturated calomel electrode (SCE) along with a salt bridge full of saturated potassium chloride solution as the reference electrode and a graphite plate (1 cm^2^) as the working electrode. Prior to the test, the working electrode was manually polished with SiC grit paper and then washed by ultrasonic cleaning in ethanol and secondly in distilled water for 10 min, respectively. The sinusoidal excitation voltage applied to the cells in the EIS test was 5 mV with a frequency range between 0.01 Hz and 100 kHz.

### 2.4. Charge–discharge tests

The charge–discharge tests were performed in a VRFB single dynamic cell, which was assembled by employing two pieces of PAN-based graphite felt (25 cm^2^) as an electrode, Nafion 117 ion exchange membrane as a separator and two copper plates as current collectors. 30 ml 1.6 M V(IV) in 2.8 M H_2_SO_4_ served as a negative electrolyte and 30 ml 1.6 M V(III) in 2.8 M H_2_SO_4_ as a positive electrolyte were cyclically pumped into the corresponding half-cell, by two peristaltic pumps with a flow rate of 10 ml min^−1^. Before the experiment, the Nafion 117 ion-exchange membrane was soaked in the 3% hydrogen peroxide solution for 1 h, then soaked in 1 mol l^−1^ H_2_SO_4_ for 30 min at 80°C and rinsed with deionized water. Each piece of graphite felt was soaked in an ethanol water solution (70%) under ultrasonication for 30 min to remove impurities in the fibres, then rinsed with deionized water and dried in an oven at 50°C for 10 h. The galvanostatic charge–discharge test of the single cell was carried out using CT2001B-5 V/10A (Wuhan Land Co., China) between 0.65 and 1.65 V at a current density of 40 mA cm^−2^.

## 3. Results and discussion

### 3.1. Effect of Fe(III) on thermal stability of V(V)

As shown in table 1, the thermal stability of the V(V) electrolyte decreased greatly when the temperature increased from 25°C to 50°C due to the endothermic nature of the precipitation reaction of V(V) ions (1) [25]:

3.1 $${2\mathrm{VO}}_{2}^{+}+H_{2}O\to V_{2}O_{5}+2H^{+}\;\Delta H>0.$$

**Figure 1.**
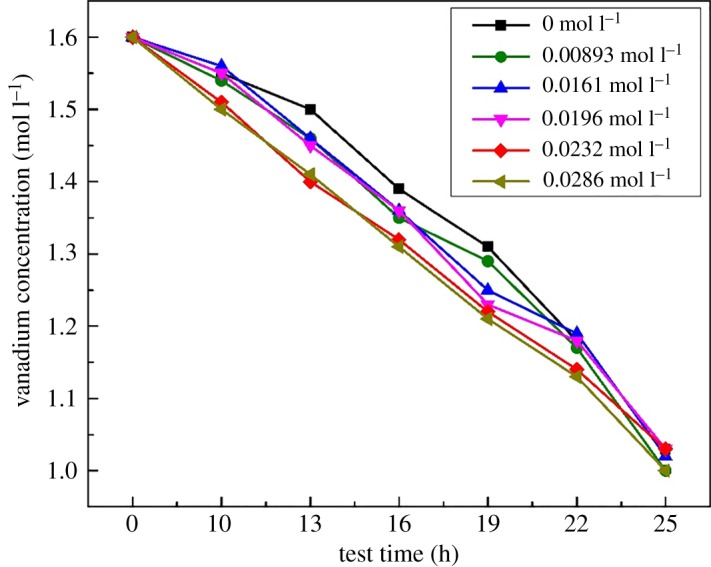
Effect of Fe(III) on the thermal stability of V(V) electrolyte at 50°C.

As observed, Fe(III) had a slight effect on the time taken for the precipitation formation. The V(V) electrolyte with different concentrations of Fe(III) could remain stable at 25°C for above 30 days. However, when the Fe(III) addition was more than 0.0196 mol l^−1^, the V(V) electrolyte could only exist stably at 40°C for 96 h; this was shorter in comparison to the pristine one which could remain stable for 108 h. The vanadium concentration of the electrolytes with different amounts of Fe(III) after a slight precipitation appeared to remain at about 1.1 M at 40°C and 1.0 M at 50°C. But the downtrend of vanadium concentration for electrolyte was faster than the pristine one when the Fe(III) addition was more than 0.0196 mol l^−1^, as shown in figure 1. The concentration of the V(V) ion decreased gradually with the prolonged heating time, and the concentration of remaining V(V) ions in the electrolyte with 0.0232 mol l^−1^ Fe(III) impurity was 0.1 mol l^−1^ lower than that of the pristine electrolyte between 10 and 22 h, indicating that when the Fe(III) impurity content was above 0.0196 mol l^−1^ it significantly impaired the thermal stability of the V(V) electrolyte.

**Table 1.** Effect of different concentrations of Fe(III) on the thermal stability of the electrolyte at different temperatures.

	time to precipitation			vanadium concentration of solution after test (mol l^−1^)	
solution (mol l^−1^)	25°C [d]	40°C [h]	50°C [h]	40°C	50°C
0	>30	108	18	1.32	1.05
0.00893	>30	108	18	1.10	1.02
0.0161	>30	108	18	1.14	1.01
0.0196	>30	120	20	1.40	0.98
0.0232	>30	96	18	1.10	1.01
0.0286	>30	96	18	1.00	1.02

1 [d] = 24 [h].

### 3.2. Cyclic voltammetry

**Figure 2.**
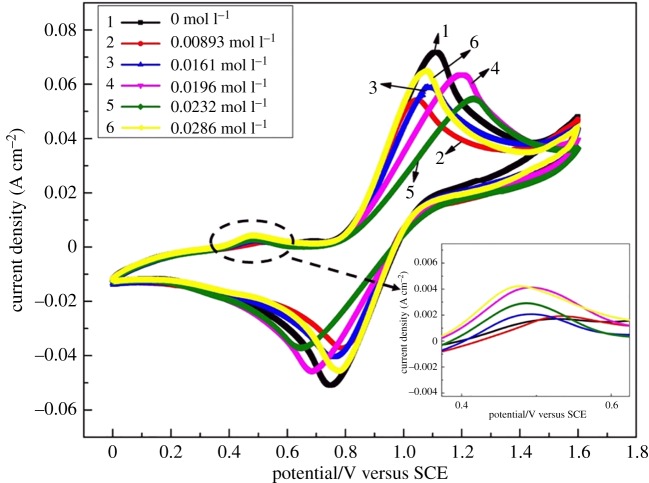
CV curves of 1.6 M VOSO_4_ at the scan rate of 10 mV s^−1^ with different concentrations of Fe(III).

Figure 2 displayed the CV curves of 1.6 M V(IV) in 2.8 M H_2_SO_4_ electrolyte with different concentrations of Fe(III) at a scan rate of 10 mV s^−1^. For convenience of comparison, the related data, shown in figure 2, are summarized in table 2. As can be seen, the electrochemical activity of the V(V)/V(IV) redox couple with different concentrations of Fe(III) was changed. As the Fe(III) concentration increased from 0.00893 mol l^−1^ to 0.0286 mol l^−1^, the Δ*E*_p_ were clearly increased and the *J*_pa_/*j*_pc_ first decreased and then increased. When the Fe(III) concentration was 0.0196 mol l^−1^, the Δ*E*_p_ was smaller and the *J*_pa_/*j*_pc_ was closer to 1 compared with the other samples, which improved the reversibility of the V(V)/V(IV) redox couple to some extent [26–28].

**Table 2.** Main data from CV curves of 1.6 M VOSO_4_ with different concentrations of Fe(III).

	anodic peak		cathodic peak			
solution (mol l^−1^)	*j*_pa_ (mA cm^−2^)	*E*_pa_ (V)	*j*_pc_ (mA cm^−2^)	*E*_pc_ (V)	Δ*E*_p_ (V)	*J*_pa_/*j*_pc_
0	71.73	1.108	51.54	0.751	0.357	1.39
0.00893	53.99	1.045	37.21	0.789	0.256	1.45
0.0161	58.56	1.083	40.64	0.758	0.325	1.44
0.0196	63.66	1.199	46.14	0.689	0.510	1.38
0.0232	54.60	1.240	37.43	0.646	0.594	1.46
0.0286	64.99	1.075	45.92	0.772	0.303	1.42

**Figure 3.**
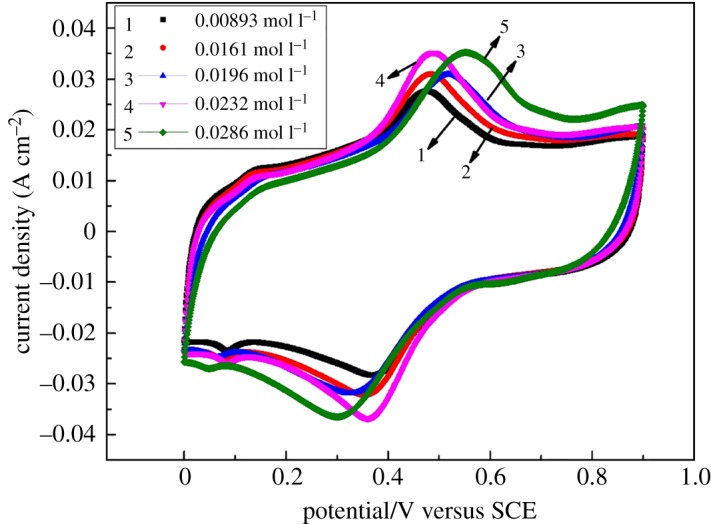
CV curves of 2.8 M H_2_SO_4_ solution with different concentrations of Fe(III) at a scan rate of 0.1 V s^−1^.

**Figure 4.**
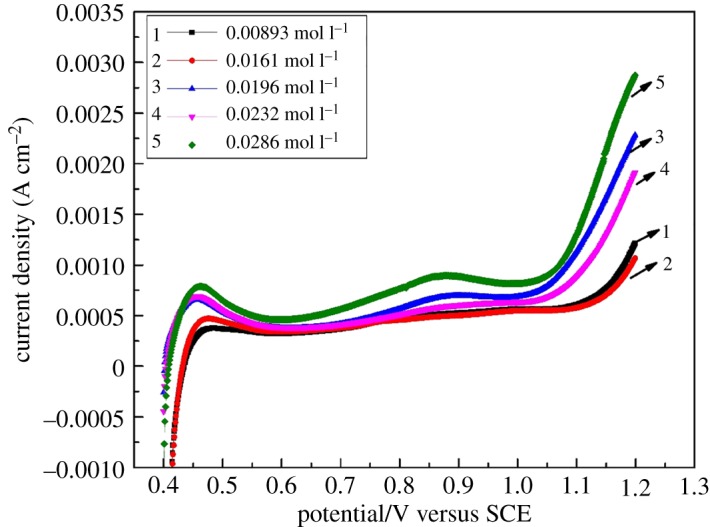
LSV curves of 2.8 M H_2_SO_4_ solution with different concentrations of Fe(III) at a scan rate of 0.001 V s^−1^.

On further study on the CV curves, a secondary oxidation peak was found at 0.4–0.6 V, as shown in figure 2. The peak currents were found to increase with the increased concentration of Fe(III). This is probably attributed to the Fe(III)/Fe(II) couple reaction. Figure 3 displays the CV curves of 2.8 M H_2_SO_4_ solution with different concentrations of Fe(III) at a scan rate of 0.1 V s^−1^. It clearly shows that iron ions underwent oxidation reaction and then formed an oxidation peak at 0.4–0.6 V. Figure 4 shows the LSV curves of 2.8 M H_2_SO_4_ solution with different concentrations of Fe(III) at a scan rate of 0.001 V s^−1^. It can be observed that the current density was increased with the concentration of Fe(III) at 1.0 V, which is the redox potential of V(IV)/V(V), so it illustrated that iron ions perhaps are competitive with vanadium ions for the adsorption on the electrode surface and redox reaction. The side reaction of the Fe(III)/Fe(II) may have a bad influence on the performance of VRFB.

**Figure 5.**
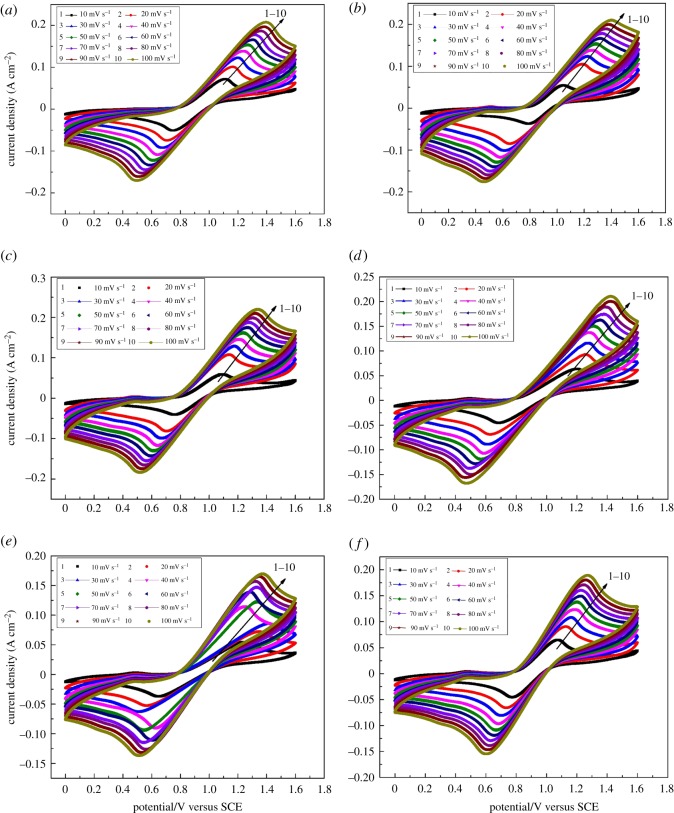
CV curves of the electrolyte (1.6 M V(IV)/2.8 M H_2_SO_4_) on graphite electrode at room temperature at different scan rates. (*a*) Blank; (*b*) with 0.00893 mol l^−1^ Fe(III); (*c*) with 0.0161 mol l^−1^ Fe(III); (*d*) with 0.0196 mol l^−1^ Fe(III); (*e*) with 0.0232 mol l^−1^ Fe(III); (*f*) with 0.0286 mol l^−1^ Fe(III).

**Figure 6.**
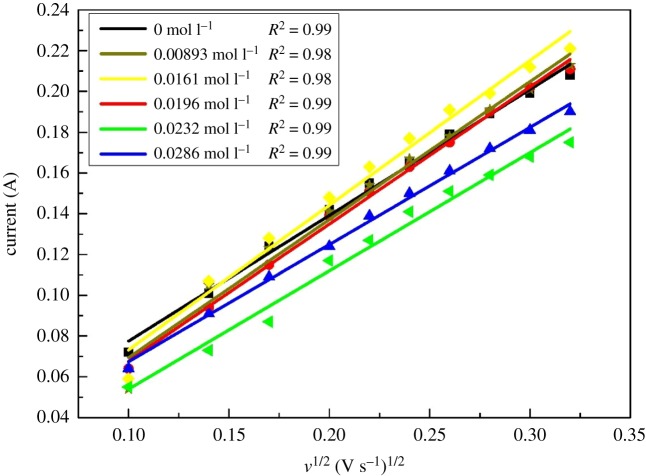
Plots of the anodic peak current versus the square root of scan rates (*υ*^1/2^) for the electrolytes with different concentrations of Fe(III).

To further investigate the effect of Fe(III) on the kinetics of electrode reaction, a series of CV curves for test electrolyte, containing different concentrations of Fe(III) on the graphite electrode at various scan rates, are shown in figure 5. It presents the typical characteristics of a quasi-reversible one-electron process for the anodic and cathodic peak potentials that change gradually with the scanning rates. A plot of redox peak currents as a linear function of the square root of scan rates with different concentrations of Fe(III) further verified the quasi-reversible process for the V(V)/V(IV) redox reaction, as shown in figure 6.

Theoretically, the value of diffusion coefficient for a quasi-reversible reaction (D) is between that for a reversible (D_1_) one and for an irreversible (D_2_) one. For a reversible and irreversible one-step and one-electron reaction, the peak current *i_p_* is given in equation (3.2) and equation (3.3), respectively [25–28]:

For a reversible reaction:

3.2 $$i_{p}=2.69\times 10^{5}n^{3/2}{\mathrm{ACD}}_{1}^{1/2}v^{1/2}.$$

For an irreversible reaction:

3.3 $$i_{p}=2.99\times 10^{5}n^{3/2}\alpha^{1/2}{\mathrm{ACD}}_{2}^{1/2}v^{1/2},$$

where *n* is the number of electrons transferred in the reaction, A is the surface area of the working electrode (1 cm^2^), C is the bulk concentration of the primary reactant, D_1_ and D_2_ are the diffusion coefficients for a reversible reaction and an irreversible reaction, respectively, and *υ* is the scanning rate.

The corresponding diffusion coefficients of the electrolyte with different concentrations of Fe(III) obtained from equation (3.2) and equation (3.3) are listed in table 3. It is clearly shown that the diffusion coefficient of V(IV) species increased from (2.06–3.33) × 10^−6^ cm^2^ s^−1^ to (2.44–3.95) × 10^−6^ cm^2^ s^−1^ with 0.0196 mol l^−1^ Fe(III), while it decreased from (2.06–3.33) × 10^−6^ cm^2^ s^−1^ to (1.78–2.88) × 10^−6^ cm^2^ s^−1^ with 0.0286 mol l^−1^ Fe(III), indicating an improvement in the diffusion of V(IV) ions when the Fe(III) concentration was below 0.0196 mol l^−1^.

**Table 3.** The diffusion coefficient of V(IV) species in electrolyte with different concentrations of Fe(III).

sample (mol l^−1)^	0	0.00893	0.0161	0.0196	0.0232	0.0286
*D*_1_ × 10^−6^(cm^2^ s^−1^)	2.06	2.47	2.72	2.44	1.82	1.78
*D*_2_ × 10^−6^(cm^2^ s^−1^)	3.33	4.00	4.40	3.95	2.94	2.88

### 3.3. Electrochemical impedance spectroscopy

**Figure 7.**
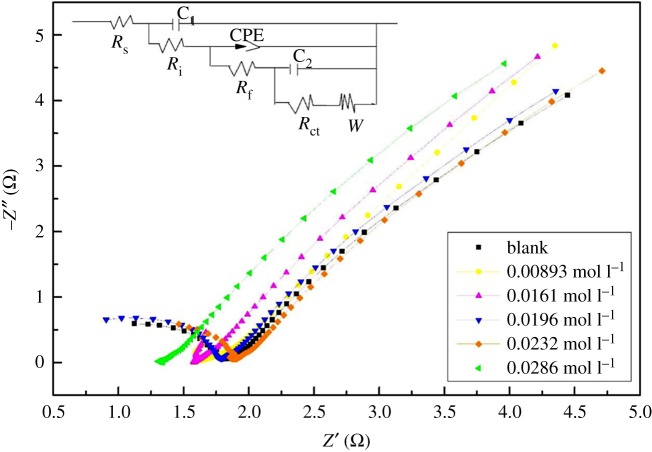
Nyquist spectra of electrochemical impedance spectra of the electrolyte with different concentrations of Fe(III).

Figure 7 shows the Nyquist plots of the electrolytes with different concentrations of Fe(III), in order to analyse the electrode reaction–diffusion kinetics of V(IV) species and the processes of mass transfer and charge transfer for the V(V)/V(IV) redox couple. Each plot consisted of a semicircle in the high-frequency region and a sloped line in the low-frequency region. The semicircle in the high-frequency region represents the charge transfer process and the straight line in the low-frequency region represents the diffusion limited process [18,25,26]. Consequently, an equivalent circuit model [18] is proposed in figure 7, where *R*_s_, *R*_i_, *R*_f_, *R*_ct_ and *W* are the solution resistance, the interface resistance, the electrolyte film resistance on the electrode surface, the charge transfer resistance and the Warburg diffusion impedance in the electrochemical process, respectively. CPE, the constant phase element, represents the electric double-layer capacitance of the electrode–solution interface. The simulation results obtained by the ZSimpWin software from fitting the impedance plots with the equivalent circuit model in figure 7 are shown in table 4.

**Table 4.** Parameters resulting from fitting the impedance plots with the equivalent circuit model.

sample (mol l^−1)^	*R*_s_ (*Ω*^.^cm^2^)	*R*_i_ (*Ω*^.^cm^2^)	*R*_f_ (*Ω*^.^cm^2^)	*R*_ct_ (*Ω*^.^cm^2^)	*W*, *Y*_0_ (S s^−5^ cm^−2^)
0	0.63	1.16	0.69	13.11	3.33 × 10^7^
0.00893	1.66	3.46	1.35	20.94	2.46 × 10^6^
0.0161	1.61	3.37	1.22	11.83	3.48 × 10^−4^
0.0196	0.73	1.36	0.96	11.14	4.63 × 10^−5^
0.0232	0.98	2.05	1.11	13.32	2.87 × 10^9^
0.0286	1.33	3.58	1.67	16.75	1.02 × 10^13^

As observed in figure 7, the semicircle in the high-frequency region had a significant change compared with the Nyquist plots of the pristine electrolyte. This indicated that the Fe(III) has a great influence on the charge transfer process. Table 4 shows that there were significant differences in *R*_s_, *R*_i_, *R*_f_, *R*_ct_ for each sample and the electrolyte was above 0.0196 mol l^−1^ Fe(III) it exhibited a higher solution resistance, interface resistance, electrolyte film resistance and charge transfer resistance than the pristine one, which indicated that the processes of mass transfer and charge transfer were more difficult after adding too large amounts of Fe(III). But the *R*_s_, *R*_i_, *R*_f_, *R*_ct_ and Warburg impedance *W* showed a minimum value when adding 0.0196 mol l^−1^ Fe(III), which indicated the 0.0196 mol l^−1^ Fe(III) enhanced the electrochemical activity and the diffusivity of vanadium ions. However, the electrochemical reaction and the vanadium ion diffusion became difficult due to the increased electrolyte film resistance and interface resistance resulting from too large amounts of Fe(III). In brief, below 0.0196 mol l^−1^, Fe(III) had positive effects on the charge transfer reaction, and the vanadium ion diffusivity, which could promote the electrode's reaction.

### 3.4. Charge–discharge test

**Figure 8.**
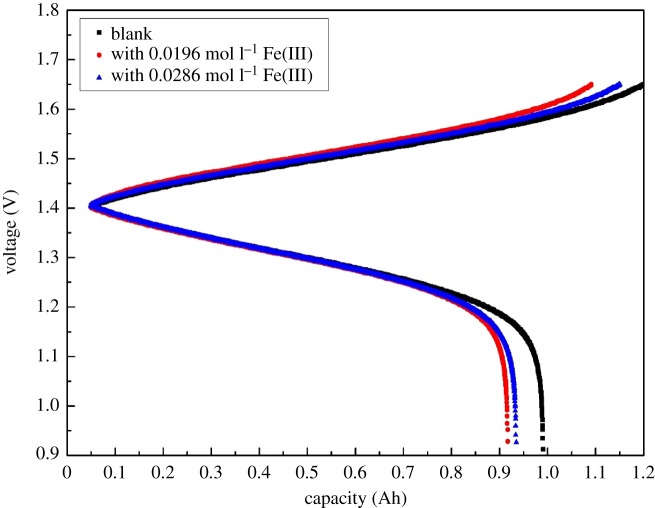
Charge–discharge curves of VRFB employing positive electrolytes with different concentrations of Fe(III) at the discharge current density of 40 mA cm^−2^.

**Figure 9.**
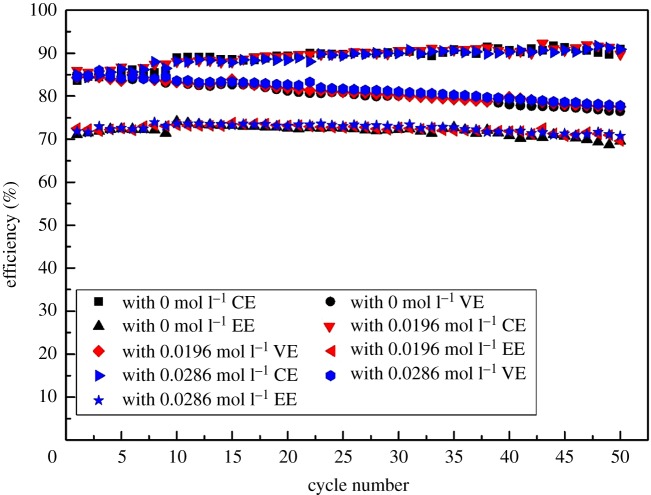
Efficiency of batteries employing positive electrolytes with different concentrations of Fe(III) at a current density of 40 mA cm^−2^.

The charge–discharge experiments were performed in a battery using a positive electrolyte with different concentrations of Fe(III) to investigate the changes of performance for the VRFB. Figure 8 shows the charge–discharge curves of VRFB with different concentrations of Fe(III) at the discharge current density of 40 mA cm^−2^. VRFB without Fe(III) presents the capacity of 0.99 Ah, which is larger than that (0.92 Ah) with the Fe(III) concentration of 0.0196 mol l^−1^ and that (0.93 Ah) with the Fe(III) concentration of 0.0286 mol l^−1^. The reason for the difference might be that Fe(III) is competitive with vanadium ions for the adsorption on the electrode surface and redox reaction, which results in the side reaction and capacity fading. Figure 9 shows the coulombic efficiency (CE), voltage efficiency (VE) and the energy efficiency (EE) of VRFB with different concentrations of Fe(III) as a function of cycling number. It can be observed that the CE, VE and EE of VRFB without Fe(III) could reach 89.10%, 80.77% and 71.92% which are little different from VRFB with 0.0196 mol l^−1^ Fe(III) (89.35%, 81.07% and 72.40%) and VRFB with 0.0286 mol l^−1^ Fe(III) (88.99%, 81.61% and 72.59%). Overall, the electrolyte with Fe(III) has little effect on the efficiency of VRFB, but Fe(III) could result in the side reaction and capacity fading, which will have a negative effect on the practical application of the battery.

### 3.5. Mechanism discussion

In view of the results discussed above, it was shown that Fe(III) has an influence on the vanadium ion diffusion, the electrode interface behaviour, the charge transfer reaction, the electrolyte impedance and the battery performance. The electrode process of V(V)/V(IV) redox reaction mainly includes [29–32]: The C-OH functional groups behave as active sites for the oxidation of VO^2+^ on the surface of the electrode. The first step in the reaction involves an ion exchange process between VO^2+^ ions transported from the bulk of the catholyte and the H^+^ ions of the phenolic functional groups on the carbon surface. Next, an oxygen atom is transferred from the C–O functional group to the VO^2+^ ions to form VO_2_^+^ on the surface of the electrode, while an electron is transferred from VO^2+^ to the electrode along the C–O–V bond. In the final step, the oxidation reaction is terminated by an ion exchange between the VO_2_^+^ formed on the electrode surface and the H^+^ ion in the electrolyte. During the discharge, the redox reactions occur in the opposite directions.

**Figure 10.**
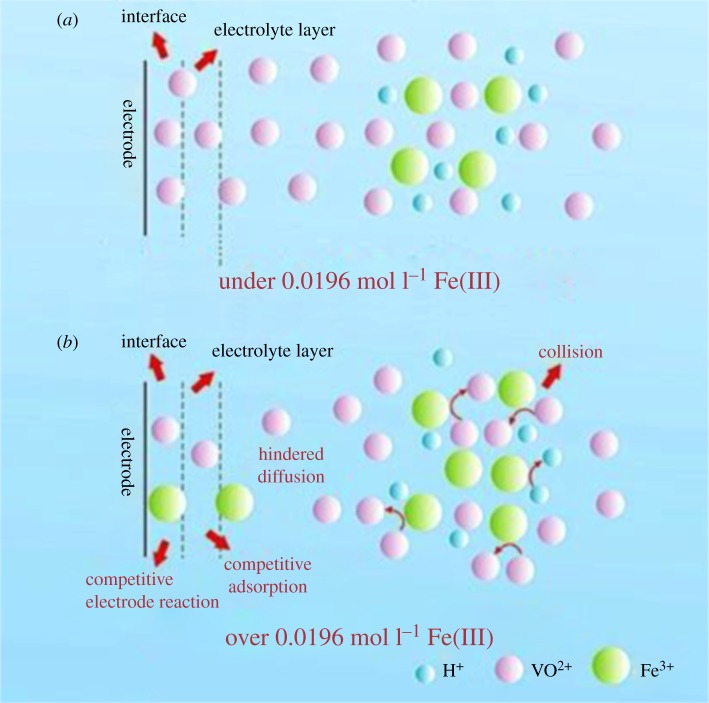
The possible mechanism for the influence of Fe(III) on the electrode process of V(V)/V(IV) redox reaction.

Therefore, the possible reasons for the influence of Fe(III) on the electrode process of V(V)/V(IV) redox reaction are as follows, as illustrated in figure 10.

#### 3.5.1. Collision and competition

The collision probability of vanadium ions with Fe(III) increases due to the larger radius of Fe^3+^ (0.63 Å) [33] compared to that of V^4+^ (0.46 Å), V^5+^ (0.355 Å) [34]. Thus, the diffusion resistance of vanadium ions increased when the concentration of Fe(III) was above 0.0196 mol l^−1^ in anolyte. Furthermore, the valence of Fe(III) is lower than that of vanadium ions; with the Fe(III) addition, the electrode surface status changes slightly and Fe(III) is competitive with vanadium ions for the adsorption on the electrode surface and redox reaction, which results in the charge imbalance on the electrode surface and alters the electric double-layer at the interface. Therefore, the electrolyte film resistance and the interface resistance increased with the addition of Fe(III). The increase of the collision probability and the competition charge–discharge can interfere with the electrode reaction, the diffusion of vanadium ions and the performance of VRFB. When the electrolyte has a lower concentration (below 0.0196 mol l^−1^) of Fe(III), the traces of Fe(III) may facilitate dispersion of vanadium ions for the synergy of coulombic repulsion and steric hindrance [25,26] and may interfere in the association of vanadium ions to a certain degree, which results in the corresponding improvement of electrode reaction activity.

## 4. Conclusion

The thermal stability tests showed that the Fe(III) impurity content above 0.0196 mol l^−1^ significantly impaired the thermal stability of the V(V) electrolyte. The CV measurements indicated that better reaction kinetics were achieved by adding 0.0196 mol l^−1^ Fe(III), resulting in the improvement of the V(IV) diffusion coefficient, and better reversibility of the electrode reaction compared with the pristine electrolyte. The EIS tests showed that positive electrolyte exhibited a higher electrolyte resistance and charge transfer resistance when the Fe(III) impurity concentration was above 0.0196 mol l^−1^. The charge–discharge tests showed that positive electrolyte with Fe(III) has little effect on the efficiency of VRFB, but Fe(III) could result in the side reaction and capacity fading, which will have a detrimental effect on battery performance.

Moreover, when the concentration of Fe(III) was above 0.0196 mol l^−1^, the increase in the collision probability of vanadium ions with Fe(III) and Fe(III) is competitive with vanadium ions for the adsorption on the electrode surface and redox reaction can interfere with the electrode reaction, the diffusion of vanadium ions and the VRFB performance.

As discussed above, understanding the influence of Fe(III) on the positive electrolyte is important, and it is best to control the concentration of Fe(III) impurity below 0.0196 mol l^−1^ during the preparation and use of vanadium electrolyte.

## Supplementary Material

test record

## Permission to carry out fieldwork

No permissions were required prior to conducting our research.

## Data accessibility

The datasets supporting this article have been uploaded as part of the electronic supplementary material.
