# Supplementary material for: Effect of Fe(III) on the positive electrolyte for vanadium redox flow battery
Source: R Soc Open Sci. 2019 Jan 23;6(1):181309. doi: 10.1098/rsos.181309 (PMC6366179; doi:10.1098/rsos.181309)
Supplement: test record [file rsos181309supp1.docx]

**Effect of Fe(III) on the positive electrolyte for vanadium redox flow battery**

Muqing Ding^a,b,c,d^, Tao Liu^a,b,c,d*^, Yimin Zhang^a,b,c,d,e^, Zhenlei Cai ^a,b,c,d^, Yadong Yang ^a,b,c,d^, Yizhong Yuan ^a,b,c,d^

*^a^ School of Resource and Environmental Engineering,* *Wuhan University of Science and Technology, Wuhan 430081, Hubei Province, China*

*^b^ State Environmental Protection Key Laboratory of Mineral Metallurgical Resources Utilization and Pollution Control, Wuhan University of Science and Technology, Wuhan 430081, Hubei Province, China*

*^c^ Hubei Provincial Engineering Technology Research Center of High Efficient Cleaning Utilization for Shale Vanadium Resources, Wuhan University of Science and Technology, Wuhan 430081, Hubei Province, China*

*^d^ Hubei Collaborative Innovation Center for High Efficient Utilization of Vanadium Resources, Wuhan University of Science and Technology, Wuhan 430081, Hubei Province, China*

*^e^ School of Resource and Environmental Engineering, Wuhan University of Technology, Wuhan 430070, Hubei Province, China*

*Corresponding Authors: E-mail addresses: [tkliutao@126.com](mailto:tkliutao@126.com) (T. Liu).

## 2.2 Thermal stability experiment

The composition and morphology of the precipitates obtained from V(V) electrolyte during thermal stability experiment were characterized with X-ray diffraction (XRD) and scanning electron microscopy (SEM). The XRD analysis was conducted using a Rigaku D/MAX 2500PC X-ray diffraction (Rigaku, Japan) using Cu Kα radiation. A JSM-6610 SEM (JEOL, Japan) equipped with an energy disperse X-ray spectrometry (EDS, Bruker, Germany) was employed.

The crystal structure of precipitates was determined by XRD. Figure 1 shows the spectrum of precipitates in the V(V) electrolyte almost corresponds to the standard spectrum of V_2_O_5_•1.6H_2_O and V_2_O_5_, suggesting that Fe(III) impurity ions did not form coprecipitates with V(V) ions. The elemental distribution of precipitates is shown in Figure 2. It shows that the atom percent of Fe and S at the pointed area A, B and C were negligible, indicating that insignificant amounts of Fe were entrapped during vanadium precipitation. The morphology of the precipitates was investigated by SEM as shown in Figure 2. Clearly, compared with the calcined precipitates extracted from the electrolyte with over 0.0196 mol L^-1^ Fe(III), the particle sizes of pristine one and the precipitates from the electrolyte with 0.0196 mol L^-1^ Fe(III) were smaller and the particles were well-distributed. The results also confirmed the excellent thermal stability of V(V) electrolyte when the Fe(III) impurity content under 0.0196 mol L^-1^, and the better thermal stability could be attributed to the enhanced dispersibility of V(V) specie.


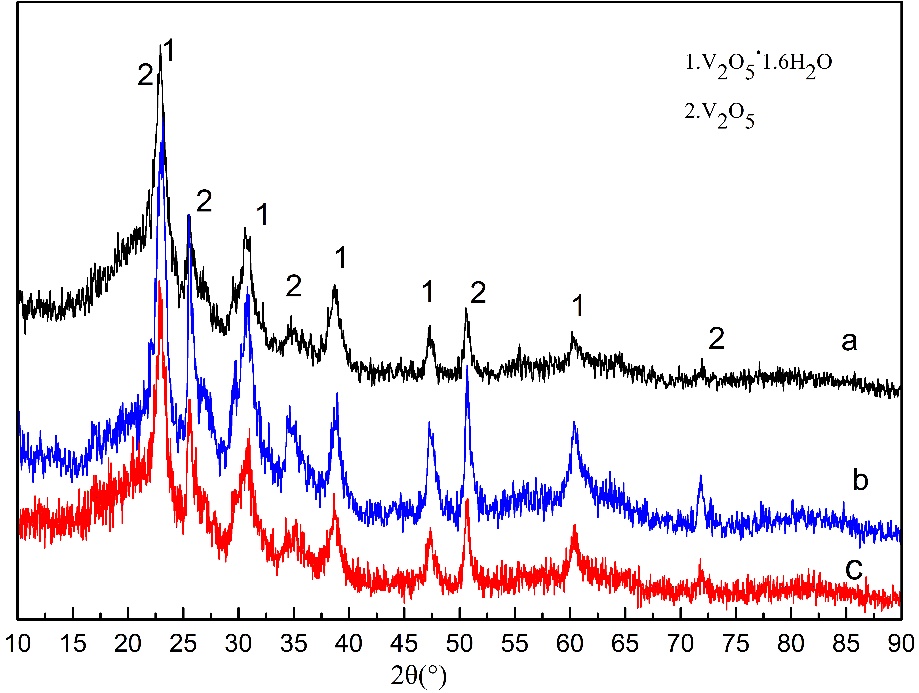


**Figure 1:** XRD patterns of precipitates extracted from the V(V) electrolyte, a. Blank; b. with 0.0196 mol L^-1^ Fe(III); c. with 0.0286 mol L^-1^ Fe(III).


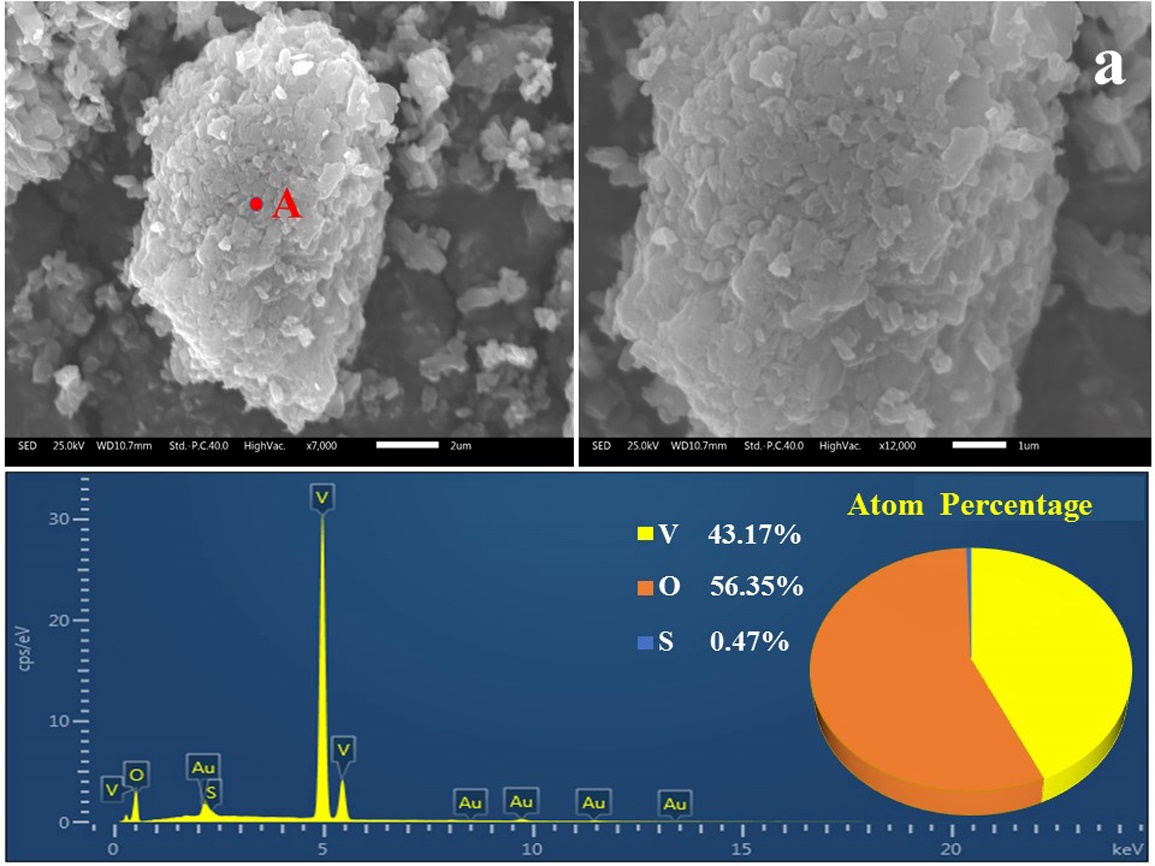


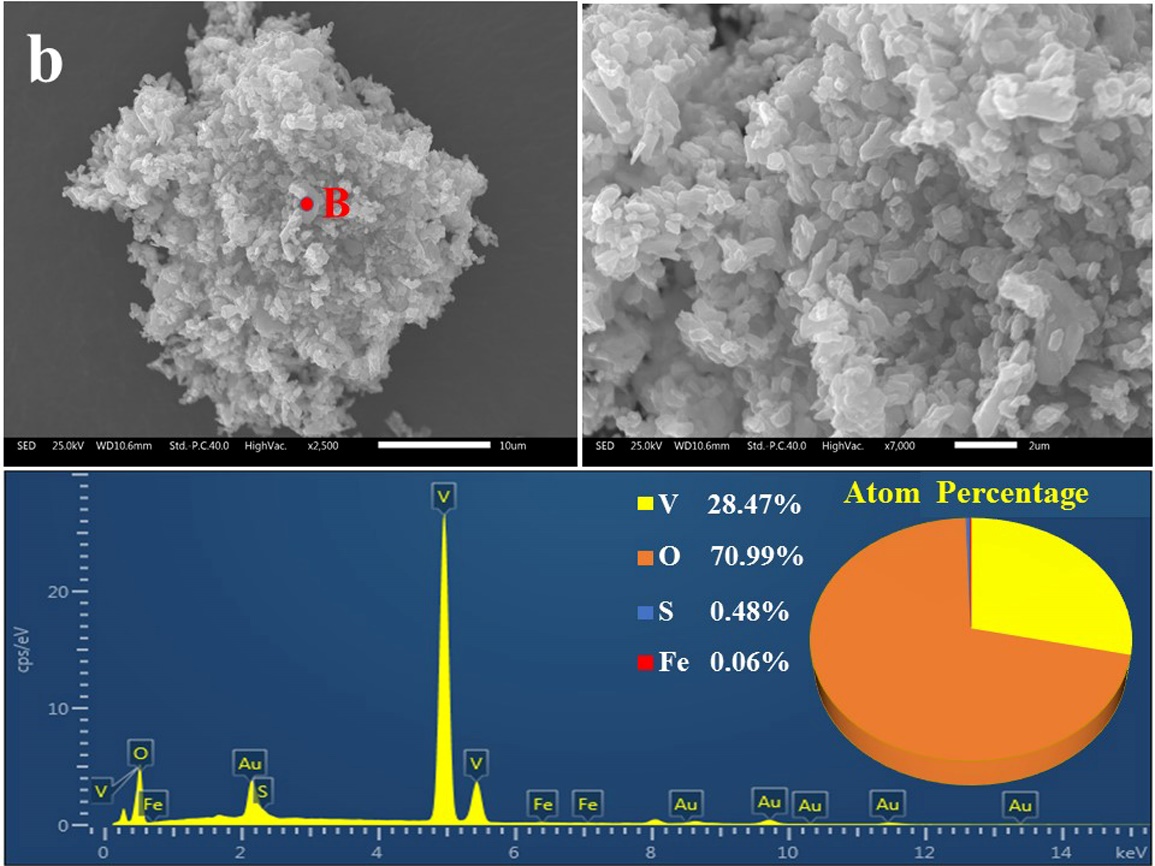


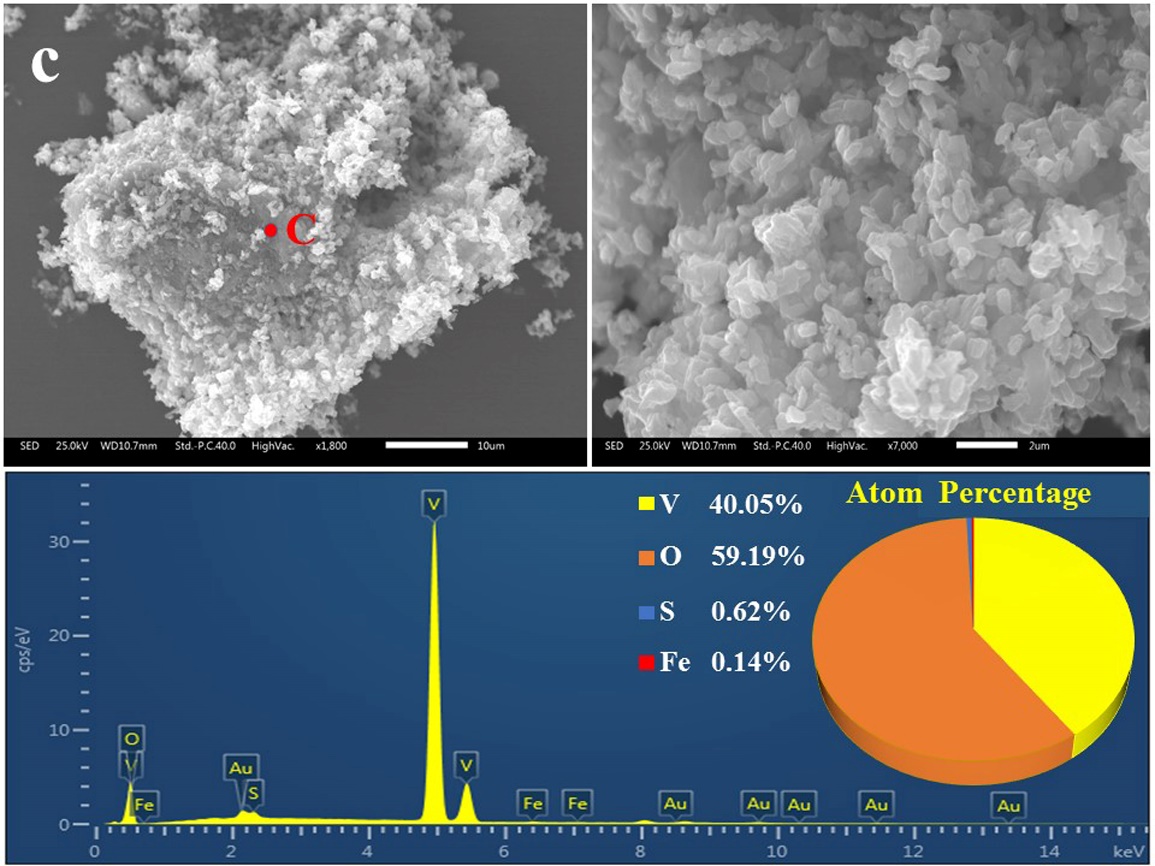


**Figure 2:** SEM images with EDS element mapping of precipitates extracted from the V(V) electrolyte, a. Blank; b. with 0.0196 mol L^-1^ Fe(III); c. with 0.0286 mol L^-1^ Fe(III).

Figure 3 showed the changes of Fe(III) concentration in the CV tests. It can be observed that the almost all of Fe(III) had transformed to Fe(II) after the CV tests.


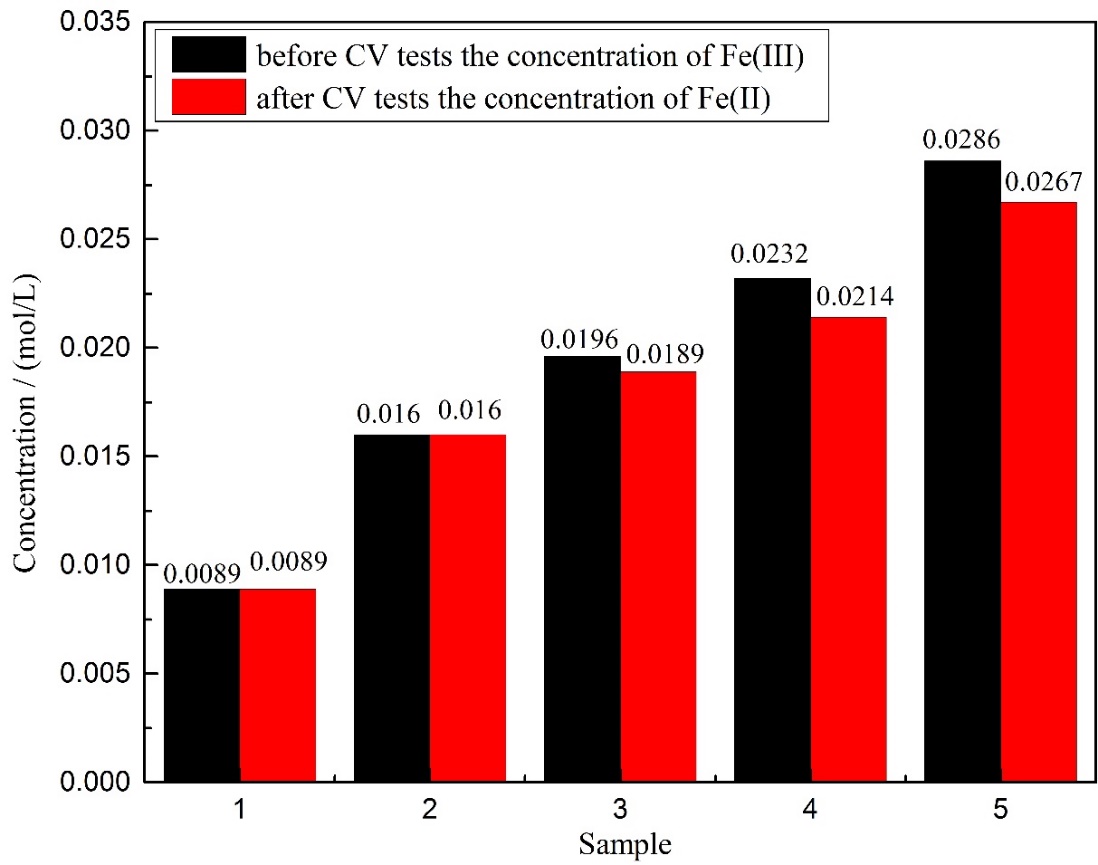


**Figure 3:** The changes of Fe(III) concentration in the CV tests.

**Results and Discussion:**

**Comment 2:** Did you measure the V(V) concentration before the measurement, and if so, what was it? What was the change? It is unclear if the “redox titration method” mentioned earlier is related to this, and it is not stated what the “redox titration method” is.

**Response:** We appreciate the reviewer a lot for the constructive comment. Before the measurement, the V(V) concentration of the test samples were 1.58 mol/L ~ 1.62 mol/L. The solutions were filtered at regular intervals and titrated to determine the changes in vanadium concentration. The downtrend of vanadium concentration for electrolyte was more quickly than the pristine one when the Fe(III) addition was more than 0.0196 mol/L, as shown in Fig.1. The concentration of V(V) ion decreased gradually with the prolonging of heating time, and the concentration of remaining V(V) ions in electrolyte with 0.0232 mol/L Fe(III) impurity was 0.1 mol/L lower than that of the pristine electrolyte between 10 and 22 hours.

The vanadium concentration in the electrolyte was analyzed by the potentiometric redox titration method. The procedure of the redox titration method is to add the sulfur and phosphorus mixed acid, then add potassium permanganate solution to the electrolyte until the solution becomes purple, and finally, have a titration using a potentiometric titrator with ferrous ammonium sulfate standard solution until the potential of electrolyte changes suddenly; the vanadium concentration can be calculated through the titration volume and concentration of standard solution [23].

[23] Y.D. Yang, Y.M. Zhang, T. Liu, J. Huang, Improved properties of positive electrolyte for a vanadium redox flow battery by adding taurine, Res. Chem. Intermed. 44 (2018) 769-786. (doi:10.1007/s11164-017-3133-y)


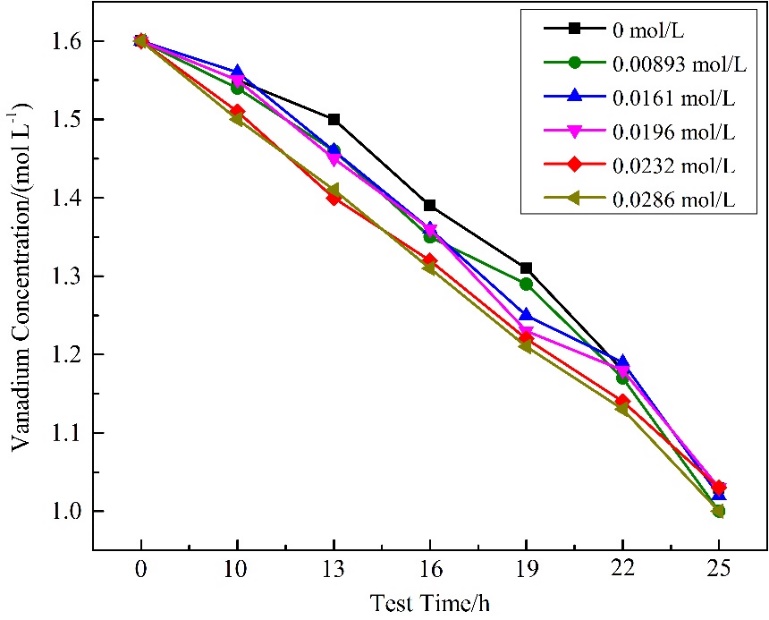


**Figure 1: Effect of Fe(III) on thermal stability of V(V) electrolyte at the temperature of 50** **°C.**

**Table 1.** Effect of different concentration of Fe(III) on the thermal stability of electrolyte at different temperatures.

| Solution  (mol L^-1^ ) | Time to precipitation | | |  | Vanadium concentration of solution after test (mol L^-1^) | |
| --- | --- | --- | --- | --- | --- | --- |
|  | 25 °C [d] | 40 °C [h] | 50 °C [h] |  | 40 °C | 50 °C |
| 0 | >30 | 108 | 18 |  | 1.32 | 1.05 |
| 0.00893 | >30 | 108 | 18 |  | 1.10 | 1.02 |
| 0.0161 | >30 | 108 | 18 |  | 1.14 | 1.01 |
| 0.0196 | >30 | 120 | 20 |  | 1.40 | 0.98 |
| 0.0232 | >30 | 96 | 18 |  | 1.10 | 1.01 |
| 0.0286 | >30 | 96 | 18 |  | 1.00 | 1.02 |

1 [d] = 24 [h]

**Table 4.** Parameters resulting from fitting the impedance plots with the equivalent circuit model.

| Sample  ( mol L^-1^ ) | R_s_  (Ω^.^cm^2^) | R_i_  (Ω^.^cm^2^) | R_f_  (Ω^.^cm^2^) | R_ct_  (Ω^.^cm^2^) | W,Y_0_  (S^.^s^-5.^cm^-2^) |
| --- | --- | --- | --- | --- | --- |
| 0 | 0.63 | 1.16 | 0.69 | 13.11 | 3.33× 10^7^ |
| 0.00893 | 1.66 | 3.46 | 1.35 | 20.94 | 2.46× 10^6^ |
| 0.0161 | 1.61 | 3.37 | 1.22 | 11.83 | 3.48× 10^-4^ |
| 0.0196 | 0.73 | 1.36 | 0.96 | 11.14 | 4.63× 10^-5^ |
| 0.0232 | 0.98 | 2.05 | 1.11 | 13.32 | 2.87× 10^9^ |
| 0.0286 | 1.33 | 3.58 | 1.67 | 16.75 | 1.02× 10^13^ |
